# Explaining Andean Potato Weevils in Relation to Local and Landscape Features: A Facilitated Ecoinformatics Approach

**DOI:** 10.1371/journal.pone.0036533

**Published:** 2012-05-31

**Authors:** Soroush Parsa, Raúl Ccanto, Edgar Olivera, María Scurrah, Jesús Alcázar, Jay A. Rosenheim

**Affiliations:** 1 CIAT (Centro Internacional de Agricultura Tropical), Cali, Colombia; 2 Grupo Yanapai, Miraflores, Lima, Peru; 3 CIP (Centro Internacional de la Papa), Lima, Peru; 4 Department of Entomology, University of California Davis, Davis, California, United States of America; The Pennsylvania State University, United States of America

## Abstract

**Background:**

Pest impact on an agricultural field is jointly influenced by local and landscape features. Rarely, however, are these features studied together. The present study applies a “facilitated ecoinformatics” approach to jointly screen many local and landscape features of suspected importance to Andean potato weevils (*Premnotrypes* spp.), the most serious pests of potatoes in the high Andes.

**Methodology/Principal Findings:**

We generated a comprehensive list of predictors of weevil damage, including both local and landscape features deemed important by farmers and researchers. To test their importance, we assembled an observational dataset measuring these features across 138 randomly-selected potato fields in Huancavelica, Peru. Data for local features were generated primarily by participating farmers who were trained to maintain records of their management operations. An information theoretic approach to modeling the data resulted in 131,071 models, the best of which explained 40.2–46.4% of the observed variance in infestations. The best model considering both local and landscape features strongly outperformed the best models considering them in isolation. Multi-model inferences confirmed many, but not all of the expected patterns, and suggested gaps in local knowledge for Andean potato weevils. The most important predictors were the field's perimeter-to-area ratio, the number of nearby potato storage units, the amount of potatoes planted in close proximity to the field, and the number of insecticide treatments made early in the season.

**Conclusions/Significance:**

Results underscored the need to refine the timing of insecticide applications and to explore adjustments in potato hilling as potential control tactics for Andean weevils. We believe our study illustrates the potential of ecoinformatics research to help streamline IPM learning in agricultural learning collaboratives.

## Introduction

Modern advances in information science, statistics, and computing power are creating unprecedented opportunities to advance agricultural science. An underexploited opportunity exists to address research questions using data routinely generated by farmers and agricultural consultants through their record-keeping activities [1]–[5]. This research approach falls under the umbrella of ecoinformatics, an emerging field in ecology that is thought to hold particular promise for integrated pest management (IPM) research [5]. There is no uniform definition for “ecoinformatics;” but its scope covers the management, integration and analysis of diverse streams of data to answer complex questions in ecology. Here, we apply an ecoinformatics approach to the joint study of local and landscape factors explaining infestations of a key potato pest in the Andes, the Andean potato weevil (*Premnotrypes* spp.).

It is widely recognized that pest impact on an agricultural field is jointly influenced by the features of that field (i.e. local features) and by features of the area surrounding it (i.e. landscape features). Seldom, however, are these local and landscape features studied jointly (but see [6]–[8]). Perhaps because they are easier to manipulate, local-level processes like host-plant resistance and chemical control are most often studied experimentally. In contrast, landscape-level processes, like the spillover of natural enemies from unmanaged areas into agricultural areas, are almost exclusively studied observationally [9]. The lack of an integrated approach results in a dearth of knowledge on the relative importance of local versus landscape-level influences and the relative payoffs of managing each.

The Andean potato weevils, a complex of tuber-boring herbivores dominated by the genus *Premnotrypes*, are the most important pests of potatoes in the high Andes [10]. They are native to the Andes, feed only on potatoes, and complete only one generation per year under traditional (rain-fed) potato agriculture [10]. Andean potato weevils appear to have reached pest status only in the past century, in response to the intensification of Andean farming systems [11]. Despite much searching, no weevil-resistant potato cultivars and only a few modestly-suppressive natural enemies (all of them generalists) have been found to date [12], [13]. An effective integrated pest management (IPM) program has been developed for these weevils [14], but adoption rates are poor due to its high labor requirements (Ortiz, personal communication).

Under rain-fed agriculture, the Andean potato weevil life cycle begins with the onset of rains (October–November), when adults emerge from their overwintering sites in soils of previous-season potato fields and potato storage facilities, and disperse by walking to find germinating potato plants [10], [15]. Most weevils disperse to find newly-planted potato fields. The remaining minority stays within previous-season fields to feed and reproduce on potato plants that re-emerge spontaneously (i.e. volunteer potatoes). A potato field's proximity to previous-season fields and storage facilities (overwintering sites) is positively correlated with infestation, while its proximity to potato fields planted during same season is negatively correlated with infestation [15]. When potatoes are reached, female adults lay eggs on the base of the plants. Upon hatching, neonate larvae dig into the soil and burrow into tubers where they feed until completing their larval life cycle. Towards the end of the potato-growing season (April–May), mature larvae begin to abandon tubers to pupate in the soil. Larvae that mature before harvest pupate in the soil of potato fields. The remaining larvae are transferred to and pupate in the dirt floors of potato storage facilities. Because volunteer potatoes are harvested and consumed by farmers early in the season (February–March), larvae within them seldom complete their life cycle (S. Parsa, personal observation).

Our objective was to jointly screen and compare the explanatory value of many local and landscape factors thought to influence Andean potato weevil populations. This task gave us an opportunity to systematically unearth and validate much knowledge on this pest reported only in the “gray” literature. We hoped this analysis would help us propose a shorter set of “priority” variables to be examined more closely by future studies seeking to refine the Andean potato weevil IPM program. We build upon a previous study that examined the explanatory importance of landscape factors [15]. This article builds on the previous analysis by evaluating the role of local factors, with data partly generated by farmers using record-keeping forms produced and distributed by our research team. We call our approach “facilitated ecoinformatics,” because farmers were included in the generation of hypotheses and accompanied in field monitoring and record-keeping activities needed to test them.

## Materials and Methods

### Ethical considerations

No specific permits were required for the described field study. Participatory work involved only adults and it was non-experimental, anonymous and voluntary. Due to high illiteracy rates, informed consent was obtained verbally. The study was designed in consultations with faculty at the University of California in Davis and it was approved by the McKnight Foundation Collaborative Crop Research Program and by regional indigenous authorities of the Chopcca Nation. No participant-identifiable data was recorded. The principles expressed in the Declaration of Helsinki were followed. The individual pictured in this manuscript has given written informed consent (as outlined in the PLoS consent form) to appear in the published photo.

### Study Site

**Figure 1:**
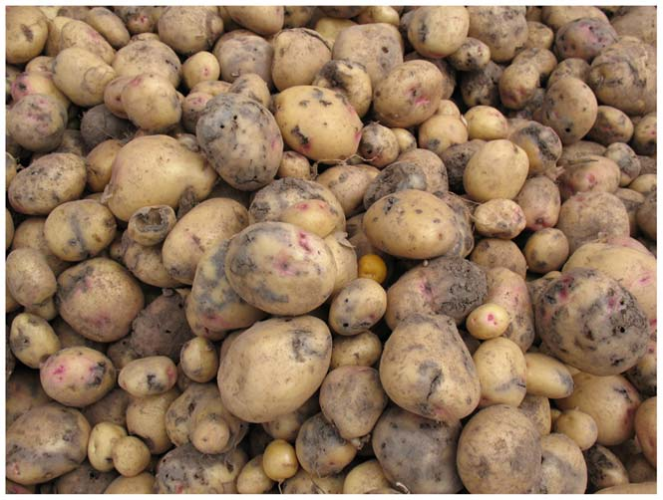
Heavy infestations by Andean potato weevils (*Premnotrypes* spp.) on improved potato cultivar Yungay (*S. tuberosum*). Photo credit: Soroush Parsa.

The study was conducted from November 2008 to May 2009 in four adjacent farming villages in the department of Huancavelica, in Peru (74°45′W, 12°46′S). The villages belong to the Chopcca indigenous nation, characterized by a traditional, subsistence-based agriculture. Potato (*Solanum* spp.) provides the main means of subsistence in the area, but it is complemented by barley (*Hordeum vulgare*), oats (*Avena sativa*), fava beans (*Vicia faba*), pearl lupine (*Lupinus mutabilis*) and minor quantities of other Andean crops. Fields are cropped once per year, with a cycle that invariably begins with potato, typically-followed by three years of non-potato cropping, and three years of fallow. Agriculture is rain-fed, with a yearly growing season that spans from October to May. As is typical for other Andean farming systems, the area is mountainous (3,500 to 4,200 meters), cold (6–12°C mean temperature) and semi-humid (500–1,000 mm/year rainfall) [16]. Farmers recognize Andean potato weevils (Fig. 1) and potato flea beetles (*Epitrix* spp.) as their most important potato pests. The dominant weevil species in the area are *P. suturicallus* and *P. piercei*. Both have similar life cycles and behavior [10]; therefore our analyses do not distinguish between the two. The potato tuber moths *Symmestrischema tangolias* and *Phthorimaea operculella* do not reach economically-important levels in the area.

### Sample of potato fields

**Figure 2:**
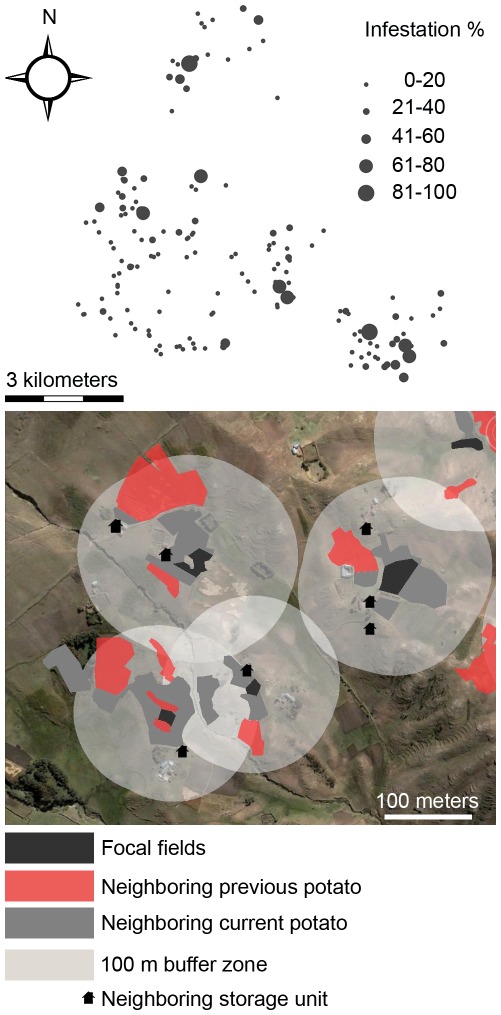
Landscape in the study area in Huancavelica, Peru. (Top) Distribution of study fields showing percentage infestation by Andean potato weevils (*Premnotrypes* spp.). (Bottom) Representative study fields showing landscape predictors of percentage infestations by Andean potato weevils.

The potato fields in this study belonged to 138 farmers randomly-selected from a roster of 643 total farmers (Fig. 2, Top). Only the cultivars Yungay (improved, *S. tuberosum*) and Larga (landrace, *S. chaucha*) were considered for the study, because they were the most abundant in the Chopcca nation and they are widely distributed in the Peruvian Andes. When a farmer had fields of both cultivars, one field was randomly-selected for the study. Data collection was coordinated by four local farmers who were extensively trained and had collaborated with us as research associates for more than a planting season. We refer to these facilitators as “community knowledge workers.” Each knowledge worker was randomly assigned to assist 20 farmers and was asked to choose roughly 15 more, both from our list of randomly-selected farmers. This flexibility allowed us to improve farmer participation rates. No farmer approached refused to participate in our study.

### Response variable

To account for edge effects on weevil distribution [15], fields were divided into edge and center sections. The outer 3 meters of the field was considered “edge,” provided it was not adjacent to (1) a barrier putatively inhibiting weevil immigration (e.g., a wall, a stream or a ditch >1 m deep) or (2) to another potato field. The remaining area was considered “center.” We measured edge and center areas using geographic positioning system (GPS) receivers (GPSMap 76CSx, Garmin Ltd., Olathe, KS) and geographic information system (GIS) software (ArcGIS, ESRI, Redlands, CA). We then sampled 20 evenly-distributed plants from each stratum (i.e., 40 per field) and inspected tubers to score for presence/absence of larvae, external bruising or emergence holes that are distinctly characteristic of weevil damage. Our response variable was the percent of tubers infested in the edge and center of fields, weight-averaged by their respective areas. Tuber infestation was scored within 10 days of the field's intended harvest date.

### Explanatory variables

We generated a comprehensive list of explanatory variables believed to influence Andean potato weevil infestations (Table 1). Most variables were selected based on (1) a review of the “gray” literature on Andean potato weevils; (2) several unstructured interviews with local farmers; and (3) a workshop of experts convened by our research team that brought together agricultural scientists and NGO practitioners working with Andean agroecosystems for more than two decades.

**Table 1:** Local and landscape features of hypothesized to exert important influences on Andean potato weevil infestations (*Premnotrypes* spp.).

Variable category	Variable description	Mean ± SD or mode
**Chemical control**	Carbofuran at planting (yes/no)	no = 106/138
	Insecticide treatments December	0.51±0.61
	Insecticide treatments January	1.12±0.74
	Insecticide treatments February	0.34±0.52
	Insecticide treatments March	0.02±0.15
**Cultural**	Ash application at planting^a^ (yes/no)	no = 134/138
	Chemical fertilization at planting^b^ (g/plant)	8.13±7.03
	Day of first potato hilling^c^ (days after Oct 1^st^)	104.10±8.57
	Harvest day^d^ (days after Oct 1^st^)	201.34±9.50
	Height of first potato hilling^c^ (cm)	18.30±3.79
	Height of row at harvest^c^ (cm)	29.02±5.17
	Manure fertilization at planting^b^ (g/plant)	75.87±21.80
	Number of hillings^c^	2 = 131/138
	Perimeter/area ratio^e^ (m^−1^)	0.26±0.10
	Planting day^f^ (days after Oct 1^st^)	48.96±8.39
	Plants/5 meters row^g^	14.81±1.74
	Rotation 2006^h^ (potato/other)	other = 114/138
	Rotation 2007^h^ (potato/other)	other = 136/138
	Row distance^g^ (cm)	95.35±11.72
	Weed removal^i^ (yes/no)	yes = 75/138
**Geographic**	Elevation^j^ (m)	3747±148.40
	Field slope^k^ (cm)	23.04±10.79
**Host related**	Potato cultivar^l^ (Yungay/Larga)	Yungay = 87/138
**Soil**	Clay^m^ (%)	21.08±5.71
	Loam^m^(%)	34.01±8.01
	Sand^m^ (%)	44.91±9.62
	K^m^ (ppm)	331.58±201.75
	P^m^ (ppm)	28.22±21.94
	Organic matter^m^(%)	5.87±2.38
	pH^m^	4.97±0.98
**Landscape**	Neighboring current potato (%)^n^	8.79±9.71
	Neighboring previous potato (%)^o^	4.08±4.32
	Neighboring storage units^p^	1.01±1.01

a Farmers apply a layer of ash directly below the potato seed at the time of planting; this practice is intended to kill potato weevils.

b Fertilization can influence crop defenses against herbivores [17].

c Many agronomists recommend hilling the plants (piling dirt up around the stem of the plant) higher to lengthen the distance weevil larvae must travel to find tubers.

d Early harvest shortens the exposure of tubers to neonate larvae [18].

e Larger fields have lower perimeter to area ratios and have been suggested to have lower infestations [19].

f Early emerging plants may experience greater infestations [20].

g Planting density may influence the abundance of many insect pests [21].

h Planting potatoes following a potato planting should lead to very high infestations [19], but implementing a single host free period should eliminate this risk. Rotation 2007 indicates if potatoes were sown in the field the previous season while Rotation 2006 indicates if potatoes were sown there two seasons before the study.

i The study hypothesized that weeds may serve as refuges for adult weevils before potato plants emerge.

j Weevils are poorly adapted to elevations above 3,700 meters [18].

k The study hypothesized that greater soil erosion in steeper slopes may increase tuber exposure to weevils.

l Modern cultivars like Yungay may be more susceptible to insect pests [22].

m The study was interested in exploring any soil influences on weevil infestations without any strong *a priori* expectations.

n A measure of potato fields sown the within 100 m of the focal potato field; these current fields dilute the effect of immigrating weevils [15].

o A measure of potato fields harvested the previous season that lie within 100 m of the focal potato field; these previous fields may be sources of overwintering weevils that immigrate into focal fields [14], [15].

p Potato storage units are facilities adjacent to farmer houses and are known to concentrate high densities of overwintering weevils that may immigrate into focal fields [14]–[15].

**Figure 3:**
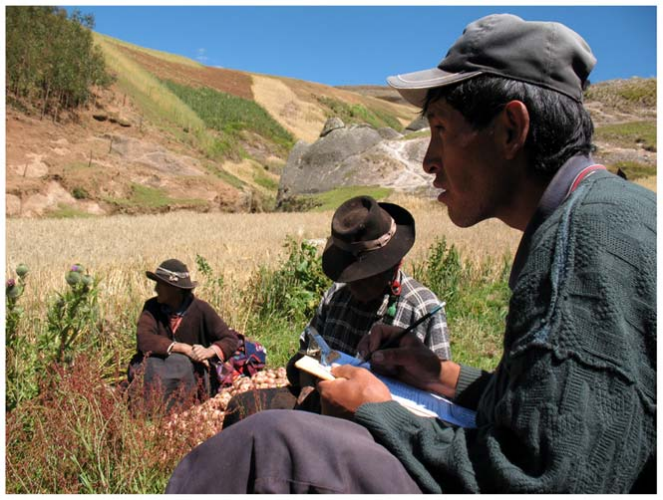
Community knowledge worker assisting farmers with record-keeping activities associated with their potato harvest. The individual pictured in this manuscript has given written informed consent (as outlined in the PLoS consent form) to appear in the published photo. Photo credit: Soroush Parsa.

Data on explanatory variables were collected combining farmer record-keeping and direct field measurements. We designed and distributed standard record-keeping forms for key management activities (e.g., pesticide applications, fertilization, weedings) to each participating farmer. To facilitate compliance with our record-keeping requirements, knowledge workers visited each farmer at least twice during the growing season, assisting them as needed to accurately fill out the forms (Fig. 3). The remaining data were gathered directly by our knowledge workers, often following their visits to farmers. Landscape and geographic data were gathered with GPS receivers and GIS software. We mapped three features within 100 m of each focal field: current potato fields, previous-season potato fields and storage units (Fig. 2, Bottom). Potato fields were expressed as the percentage of the surveyed area they occupied, whereas storage units were expressed as counts. We considered the 100-m scale adequate, because weevils only disperse by walking, they have no effective natural enemies that disperse long distances, and the area considered often contained one or more natural streams preventing their dispersal. Accordingly, landscape features separated from focal fields by streams were omitted (see [15] for details of landscape analysis). Soil data were obtained through laboratory analyses. For each field, roughly 100 grams of soil was collected from the base of each plant evaluated for weevil infestations, the samples mixed, and a consolidated sub-sample submitted to the laboratory (Laboratorios Analitícos del Sur; Arequipa, Peru).

### Statistical modeling

Analyses were conducted using JMP 7 statistical software [23]. Percent tubers infested was square-root transformed to meet assumptions of parametric statistics. We conducted two separate analyses. First, we conducted a multiple regression analysis to assess the efficacy of pesticide treatments over time. For this analysis, we regressed infestations against the number of treatments applied each month from December until March.

Second, following the information theoretic approach [24], we set out to identify the subset of explanatory variables that produced the best multiple regression model of weevil infestations. The information theoretic approach is thought to be less vulnerable to finding spurious effects due to over-fitting, and more informative than methods based on null hypothesis testing [25]. Multiple models, representing alternative explanations for the patterns observed, are compared to evaluate which one is best supported by the data. When many models are similarly-supported by the data, inferences can be made using all models considered important. In such cases, parameter estimates are averaged across models, weighted by the probability that their originating model is the best in the set. Hence, the relative importance of a parameter is not based on p-values, but rather on its occurrence in the model(s) that are best supported by the data.

To be conservative in our initial selection of variables, we first developed the least parsimonious model that was best supported by the data, as assessed by Akaike's information criterion (AIC) [24]. Variables were added to this initial model one at the time in the order dictated by their influence on the AIC (variables that reduced the AIC the most were entered first). We continued adding variables even when their addition penalized (or increased) the AIC, as long as the resulting model had an AIC value no more than 2.0 larger than the lowest AIC values reached in previous steps. We chose this threshold because models within 2.0 AIC values are thought to be similarly supported by the data [24]. Our analysis included four categorical variables to control statistically for possible observer effects associated with our four knowledge workers. Interaction terms were omitted because including them would have made the analyses too computationally intensive, and we lacked specific hypotheses linking them to weevil infestations.

We then evaluated models resulting from all possible additive combinations of the selected variables. This process identified 51 models that were within 2 AIC values of the best, indicating substantial model selection uncertainty [24]. Under these circumstances, the information theoretic approach advocates model averaging. Accordingly, three steps were followed to average estimates across the 51 models. First, we computed each model's Akaike weight (*w*_i_), which is interpreted as the relative probability that a given model is the best in the set. Second, we computed the weight for each parameter (*w*_p_), which is interpreted as the probability that the parameter is included in the best model in the set, and is obtained by summing Akaike weights across all models where the parameter occurs. Hence, the parameter weight is a measure of the relative importance of a parameter. Finally, we weight-averaged parameter estimates. To do so, we multiplied the estimates of each model where a parameter occurred by the corresponding Akaike weights for the model; the resulting products were summed; and the sum divided by the parameter weight. This computation yields a “natural” average of the parameter estimate, because it considers only those models where the parameter occurs. However, multi-model predictions also need to take into account the evidence from models where the parameter does not occur. Accordingly, a second average for the parameter estimate was derived for predictions, by multiplying the “natural” average by the corresponding parameter weight.

To assess the importance of jointly modeling local and landscape variables, we followed the procedure above to derive two additional models: (1) the best model with only local predictors and (2) the best model with only landscape predictors. Then, we computed Akaike weights for the three to estimate their relative support.

We tested for spatial autocorrelation in model residuals with the ncf package [26] for R 2.9.1 (www.r-project.org) using the correlog() function to assess autocorrelation via Moran's I index [27]. We tested for autocorrelation with a 1,000 permutation test for fields up to 3 kilometers apart at intervals of 250 meters. No evidence for spatial autocorrelation of residuals was detected. Unless otherwise stated, mean values are presented with their standard deviation.

## Results

### Observations and descriptive statistics

For each potato field, we evaluated an average of 654±241 tubers. Infestations averaged 25.1±20.9% on field “edges” and 16.1±17.4% on field “centers,” yielding a weight-averaged infestation of 18.3±18.1%. Fields were small, averaging 424.6±282.4 m^2^. Farmers who applied carbofuran at planting did so at roughly 25 kg of active ingredient per hectare. After plant emergence insecticide treatments varied little, generally consisting of applications of methamidophos with a manual backpack sprayer at roughly 534 ml (320 g) of active ingredient per hectare. Summary statistics for the explanatory variables are presented in Table 1.

### Insecticide efficacy model

**Figure 4:**
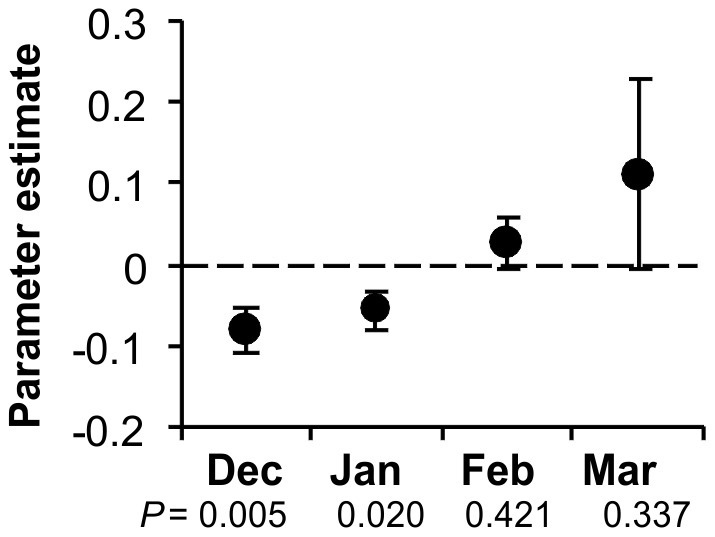
Temporal decay in the efficacy of insecticide treatments against Andean potato weevils (*Premnotrypes* spp.), as applied by farmers. The x-axis shows the parameter estimate ± SEM associated with the effect of a single insecticide application on the proportion of tubers infested with weevils (sqrt-transformed). The y-axis shows the month of the insecticide treatment.

The insecticide treatment model revealed a temporal decay in the efficacy of treatments, with treatments made after January having no significant effect on potato weevil infestation (Fig. 4)

### Development of global (least parsimonious) model

**Figure 5:**
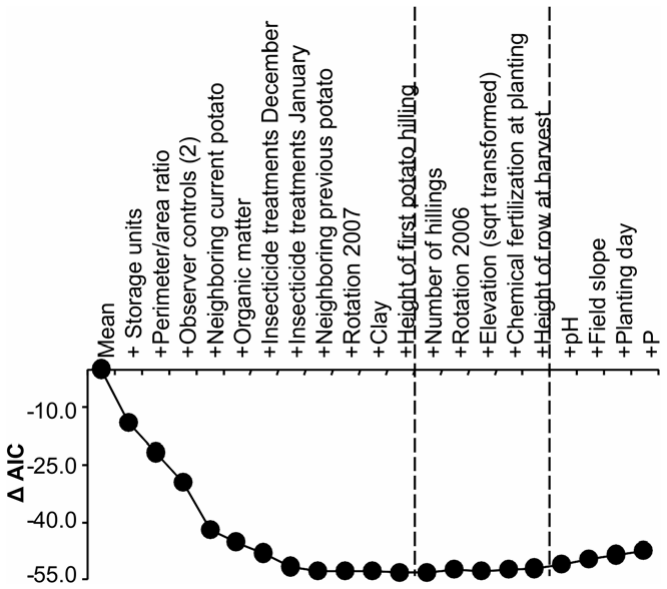
Forward stepwise development of the global (least parsimonious) statistical model explaining Andean potato weevil infestations. The x-axis shows the progressive addition of explanatory variables in order of their contributions to lowering AIC. The y-axis shows cumulative reductions in AIC from the AIC associated with using only the mean to estimate infestations. The first dashed line shows the point where the addition of variables started to penalize the AIC, whereas the second dashed line shows the point where this penalty started to exceed two AIC values. The global model included all variables before the second dashed line.

The forward stepwise development of the global model, using AIC as the criterion for variable selection, is shown in Figure 5. Because the value of the AIC has no direct interpretation (i.e. it is a comparative measure of model adequacy), our “starting point” was the AIC associated with using the mean to predict infestations, and we evaluated changes in this AIC as we added each explanatory variable. The best model derived from this method included 13 variables (2 of them control variables) and reduced the AIC by 53.3. From this point we added four more explanatory variables, which collectively penalized the best AIC by less than 2.0. Hence, the resulting global model included 15 possibly-important explanatory variables and 2 control variables (17 total variables) that collectively reduced the AIC by 52.0. We considered the remaining variables “unimportant” given the dataset.

### Model averaging

**Figure 6:**
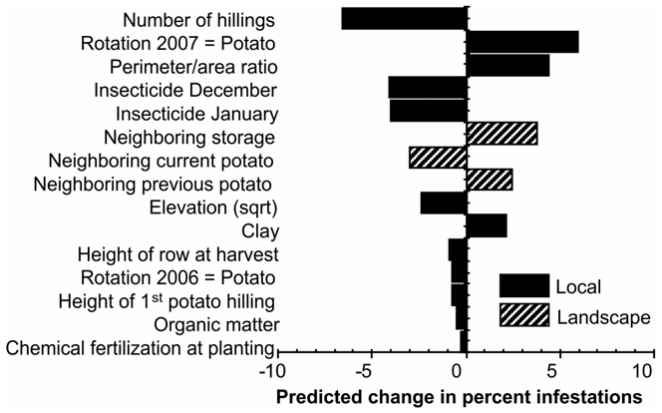
Standardized predicted impacts of explanatory variables on Andean potato infestations. The model is initially set to predict infestations for a field with no pesticide applications and with mean (for continuous variables) or most common (for ordinal and categorical variables) values for all other explanatory variables. For continuous explanatory variables, bars reflect predicted changes in infestations in response to a one standard deviation increase in the explanatory variable. For ordinal explanatory variables, the bars reflect predicted changes in infestations in response to a single unit increase in the explanatory variable; except for the number of hillings, for which only a decrease could maintain predictions within observed bounds.To obtain multi-model predictions, parameter estimates were multiplied by their corresponding parameter weights. Hence, predicted effects are “attenuated” for explanatory variables with parameter weights smaller than 1.

The combinations of 17 variables generated 131,071 total models. The top 51 models (i.e. within 2.0 AIC from the model with the lowest AIC) explained 40.2–46.4% of the variance in our dataset. Within this subset of well-supported models, none had a high probability of being the single “best” (0.036>*w*_i_>0.013); thus, all 51 models were similarly well-supported by the data. Results from averaging estimates across all 51 models are presented in table 2. All 51 models included three landscape variables (i.e., perimeter to area ratio, current potato fields, storage units), two local variables (i.e., insecticides December and January), and the controls for observer effects, as evident by their parameter weights of 1. The least commonly-included variables were chemical fertilizer and soil organic matter %, whose parameter weights were 0.23 and 0.21 respectively. The relative impact of each explanatory variable on weevil infestations is projected in Figure 6.

**Table 2:** Parameter estimates weight-averaged across the 51 “best” models predicting Andean potato weevil infestations (square root of proportion infested tubers).

			95% CI		
Variable	Estimate	SEM	Lower	Upper	*ω*_p_
Intercept	1.774	0.936	−0.061	3.608	1
Observer effect 1	−0.043	0.019	−0.081	−0.005	1
Observer effect 2	−0.070	0.016	−0.102	−0.038	1
Perimeter/area ratio	0.519	0.150	0.225	0.814	1
Neighboring storage units	0.044	0.015	0.015	0.073	1
Neighboring current potato	−0.004	0.002	−0.007	−0.001	1
Insecticide treatments January	−0.053	0.021	−0.094	−0.013	1
Insecticide treatments December	−0.054	0.025	−0.103	−0.004	1
Neighboring previous potato	0.007	0.004	0.000	0.014	0.91
Soil clay	0.005	0.003	0.000	0.010	0.91
Elevation (sqrt transformed)	−0.031	0.013	−0.057	−0.005	0.83
Number of hillings	0.127	0.076	−0.022	0.275	0.71
Rotation 2007 [Not potato]	−0.096	0.062	−0.216	0.025	0.71
Rotation 2006 [Not potato]	0.030	0.020	−0.008	0.069	0.49
Height of first potato hilling	−0.006	0.004	−0.014	0.002	0.43
Height of row at harvest	−0.005	0.003	−0.012	0.001	0.42
Chemical fertilization at planting	−0.002	0.002	−0.006	0.002	0.23
Soil organic matter	−0.012	0.008	−0.026	0.003	0.21

*Notes*: Estimates are followed by their standard errors, their 95% confidence intervals and their Akaike parameter weights (*ω*_p_). Given a set of similarly-adequate predictive models, parameter weights estimate the probability that the parameter is included in the best model in the set.

### Relative support for local, landscape, and joint models

The best models considering the influence of local and landscape factors in isolation had no probability of outperforming the model considering them jointly (Table 3).

**Table 3:** Parameter estimates ± SEM for the best models predicting Andean weevil infestations based on local factors only (i.e. Best local), landscape factors only (i.e. Best landscape) or both local and landscape factors together (i.e. Best combined).

	Best combined	Best local	Best landscape
ΔAIC relative to best combined	0	+16.03	+18.18
*w*_i_	1.00	0.00	0.00
Perimeter/area ratio	0.501±0.149**	0.548±0.158**	
Neighboring storage units	0.040±0.014**		0.048±0.015**
Neighboring current potato	−0.004±0.002**		−0.006±0.002**
Insecticide treatments January	−0.054±0.020**	−0.066±0.021**	
Insecticide treatments December	−0.051±0.025*	−0.066±0.026**	
Neighboring previous potato	0.008±0.004*		
Clay	0.006±0.003*	0.006±0.003*	
Elevation (sqrt transformed)	−0.028±0.013*		
Number of hillings	0.107±0.066	0.126±0.068	
Rotation 2007 [Not potato]	−0.083±0.060	−0.134±0.063*	
Rotation 2006 [Not potato]	0.032±0.019		
Height of first potato hilling	−0.007±0.004	−0.007±0.004	
Organic matter		−0.012±0.006	

*Notes*: Lower AIC values suggest better model performance. The Akaike weight, *w*_i_, is interpreted as the relative probability that a given model is the best in the set. The models included control variables for observer effects (not presented in the table).

* *P*≤0.05.

** *P*≤0.01.

## Discussion

Our objective was to identify a key set of variables explaining Andean potato weevil infestations in farmers' fields. We started by generating a comprehensive list of variables, including both local and landscape factors deemed important by farmers and researchers. The explanatory importance of these variables was screened statistically using an information theoretic approach, affording a simultaneous evaluation and contrast of local and landscape factors explaining weevil infestations.

Our results support the importance of studying local and landscape processes jointly. The best models considering either landscape or local factors in isolation had no probability of outperforming the best joint model. Although still rare, the number of pest management studies considering local and landscape factors jointly is increasing (e.g., [6]–[8]). To our knowledge, however, our study is the first to use crop management records as a source of local-level data.

Our findings confirm the suspected influence of some factors, fail to support the suspected influence of others, and also reveal altogether unsuspected patterns that deserve further investigation. At the local level, our results support (1) the efficacy of foliar insecticide treatments before - but not after - February, (2) the efficacy of implementing a single potato-free rotation period, and the trends of decreasing infestations with increasing (3) elevation, (4) field perimeter-to-area ratio and (5) hilling height. At the landscape level, our results support the suspected influence of (6) storage units and (7) previous-season potato fields as sources of weevil infestation. Given what is already known about Andean potato weevils, confirming these patterns is unsurprising, but it provides confidence in our results. More importantly, however, estimating these factors simultaneously for the first time affords a contrast of their predicted influences on weevil infestations. For example, multi-model estimates predict that, on average, our farmers may offset the risk of infestation from a neighboring storage with a single pesticide treatment before February (Fig. 6).

Unsupported factors included manure fertilization, planting day, planting density, row distance, weeding, slope, potato cultivar, and many soil factors. Most of these variables had been included in our study without strong *a priori* expectations. Lack of support for factors of reported importance, including several tactics farmers target at weevils, is harder to interpret conclusively. For example, our study failed to support the efficacy of carbofuran and ash treatments at planting, as well as the efficacy of foliar insecticide treatments after January. We suspect these practices are in fact ineffective. Previous observational evidence suggested that the systemic effect of carbofuran treatments at planting is lost too early in the season to be effective against the progressively-immigrating Andean potato weevils [28]. By contrast, because most weevils colonize fields before February [29], treatments in March or April should not be expected to be efficacious. A field experiment one of us conducted to test the validity of ash treatments also failed to demonstrate impacts on weevil infestations (R. Ccanto, unpublished data). These observations point to the need to address local gaps in Andean potato weevil knowledge.

Despite being one of the most widely-used preventive tactics against Andean potato weevils, we found no link between early harvest and infestations. Early harvest is thought to prevent infestations by late-hatching weevils and to intercept the development of those already within tubers before significant damage is incurred. In a post-hoc analysis, we considered the possibility that farmers with greater expected infestations were harvesting earlier. We thought this was possible, because farmers were observed to sample some of their potatoes several days before harvest to assess their maturity, and presumably also infestation levels. This could allow farmers to adjust their harvest date based on the level of infestation that they observed in their early samples of tubers. Indeed, we found that farmers with greater expected infestations, as estimated by our model, harvested earlier ([harvest date] = 207.4+−15.97*[expected infestations]; d.f. = 136, *P* = 0.01).

The previous analysis highlights the special care that must be taken when interpreting observational studies in pest management. Many management tactics are best used adaptively, i.e., in response to pest population densities observed during routine sampling. For example, if pest populations are adequately monitored and pesticides applied when needed, one might expect to find a positive association between the pest densities and the number of pesticide treatments. This adaptive behavior can eliminate what otherwise might be a negative association between the use of an effective pesticide and pest densities. We would not want to conclude from such a correlation that pesticide applications were ineffective. This particular problem did not apply to insecticide treatments in our study, because farmers were not applying pesticides adaptively (adult weevils are not monitored). However, we failed to see a correlation between infestations and harvest date presumably because farmers were adjusting their harvest date based on monitoring tuber infestations. Accordingly, observational studies in pest management demand cautious interpretation, and they are most powerful when coupled with manipulative experiments conclusively-testing variables of interest [5].

This study revealed two unsuspected results we believe deserve empirical attention. At the landscape level, we demonstrated that infestations are negatively correlated with the abundance of neighboring potato fields. The implications of this finding have been thoroughly discussed in a previous article [15]. At the local level, our results suggest that manipulating the hilling of potato plants may contribute to improving weevil management. There was a 0.84 probability that at least one variable relevant to hilling was included in the best model predicting weevil infestations (Table 2), and the effect size of the number of hillings was one of the largest (Table 2; Fig. 6). Our observations cannot elucidate exact mechanisms, but some possibilities may be explored with manipulative experiments. First, higher hilling could lengthen the distance neonate larvae need to migrate to find tubers, potentially decreasing infestations by physical isolation or by increasing exposure to mortality factors such as soil-dwelling natural enemies. Second, the height of the hilling also determines the depth of the furrow, which could inhibit the immigration of the (flightless) adult weevils, especially when heavy rains fill furrows with water. Later in the season, hilling could have a negative effect, because it could protect weevil eggs from mortality factors such as desiccation or consumption by generalist predators. This could explain why farmers who only hilled once (always early in the season) experienced lower infestations than farmers who hilled a second time (Fig. 6). If this is the case, a management tactic that targets eggs or neonate larvae before the second hilling, for example an application of entomopathogenic nematodes [30], could produce good results.

Ecoinformatics approaches may be particularly helpful during the exploratory phase of pest management research, when a key goal is to screen a large number of potentially important variables. We describe our approach as “facilitated ecoinformatics,” because farmers were included in the generation of hypotheses and accompanied in the field monitoring and record-keeping activities needed to test them. This methodological novelty adds to the literature advocating the use of local knowledge in ecological research [31]. A similar approach to research and extension, based on structured field monitoring, record-keeping, and benchmarking, has been implemented successfully to enhance productivity in facilitated learning collaboratives (e.g., [32], [33]). Based on our experience, we suspect ecoinformatics approaches can synergize these programs to streamline learning and development in agriculture.

A greater reliance on ecoinformatics in agriculture is well justified by its unique ability to generate large datasets that capture the true spatial and temporal scale of commercial agriculture [5]. As illustrated above, however, ecoinformatics datasets are observational, and therefore are poorly suited to elicit definitive causal inferences. In addition, ecoinformatics datasets may be particularly subject to observer error, potentially introduced by farmer participation in data collection. Important advances in statistical science are improving our ability to deal with these challenges [5]. We believe, however, that ecoinformatics approaches will prove most useful when used as a tool that complements traditional experimentation, rather than in competition with experimentation, to facilitate advances in agricultural science.
